# OTC Antioxidant Products for the Treatment of Cardiovascular and other Disorders: Popular Myth or Fact?

**DOI:** 10.4172/2329-6887.1000e136

**Published:** 2015-04-21

**Authors:** Mohammad A. Kaisar, Luca Cucullo

**Affiliations:** 1 Department of Pharmaceutical Sciences, Texas Tech University Health Sciences Center, Amarillo, TX 79106, USA; 2 Center for Blood-Brain Barrier Research, Texas Tech University Health Sciences Center, Amarillo, TX 79106, USA

Cardiovascular disease (CVD) is the number one cause of mortality worldwide as reported by World Health Organization (WHO), Centers for Disease Control (CDC) and American Heart Association (AHA). The role of micronutrients has been studied extensively as CVD risk minimizing intervention. Among these, dietary supplements antioxidants available over the counter are highly commercialized but scientific evidences and clinical trials supporting their use is not conclusive yet [1]. The beneficial effects of these antioxidants are focused mainly against pathogenesis of atherosclerosis, as the primary contributor to coronary artery disease and resulting cardiovascular complications. Atherosclerosis is chronic inflammatory process of deposition of fatty cholesterol filled plaque in arterial wall which narrows the diameter and obstructs blood flow down the length of coronary artery and throughout its branches. Hyperlipidemia (high HDL: LDL ratio), high blood pressure, toxins from tobacco are assumed to be the major risk factor of atherosclerosis. If plasma LDL exceeds the regulatory capacity of endothelial cells, LDL crosses the endothelial barrier and trapped in sub-endothelial space where they are susceptible for oxidation by reactive oxygen species (ROS) - superoxide anion (^·^O_2_^−^), hydroxyl radical (OH·), hydrogen peroxide (H_2_O_2_), reactive nitrogen species (RNS), nitric oxide (NO) and peroxynitrite (ONOO·) released by endothelial cells and macrophages [2]. These modified LDL stimulates endothelial cells to express various cell adhesion molecules (VCAM-I, P-selectin etc.) and chemotactic factors (monocyte chemotactic factor-1, MCP-1) for the recruitment of monocytes. Monocyte attachment is initiated by P-selectin on endothelial cell upon binding with P-selectin glycoprotein ligand-1 (PSGL-1) on the monocytes leading to rolling and prolonging the contact of monocyte on the arterial wall and initiate diapedesis across endothelial layers. Monocytes differentiate into macrophages under the influence of monocyte colony stimulating factor (M-CSF). Oxidized LDL is then engulfed in an unregulated fashion by macrophage scavenger receptor resulting in intracellular accumulation of cholesterol and forms foam cell formation [3,4]. Macrophages are also involved in activation of T cells to amplify the inflammatory response by secreting TNF-α and INF-γ. Finally, smooth muscle cells proliferate and migrate from tunica media and shield the fatty plaque and synthesize collagen to form a fibrous cap [5–7].

Despite the lack of strong biological evidences, widespread consumption of antioxidant supplements by patients suffering from cardiovascular disorders has made antioxidant therapy a multibillion industry. Among these highly advertised and frequently used antioxidants vitamin E (α tocopherol), vitamin C (ascorbic acid), co-enzyme Q-10 CoQ (10), α-lipoic acid (ALA), resveratrol and glutathione (GSH) have received highest attention due to their supposed beneficial effects in reducing the risk of CVD. However, results from randomized clinical trials are still inconclusive to support or disprove these claims [8]. Several in-vitro and in-vivo studies have demonstrated that vitamin E inhibits oxidation of LDL. The antioxidant effect is facilitated by the lipophilic nature of Vitamin E which favor its interaction with the target molecule [9]. In addition to blocking this crucial step of atherosclerosis initiation vitamin E has been proven to inhibit the progression of atherosclerotic inflammatory cascade via intracellular antioxidant effects due to its ability to cross the lipid bilayer of the cellular membranes [10,11]. Cell targets comprise vascular endothelial cells, monocyte/macrophage, platelets and smooth muscle cells. To this end vitamin E negatively modulates their inflammatory response. The downstream effect of inflammatory activity inhibition by vitamin E includes the following: 1) down regulation of the expression of cell adhesion molecules [12–14] which hinders endothelial–monocyte adhesion; 2) decreased cytokine release [15] and repression of monocytes scavenger receptor activity to engulf ox-LDL [16]; 3) inhibition of platelet aggregation [17,18] and smooth muscle cell proliferation (this latter reduced the risk of narrowing of the arterial walls) [18,19]; 4) decreased foam cell formation [20] and monocyte to macrophage differentiation by protected paraoxonase (PON 1) [21]. Detailed molecular studies suggests that the antioxidant and anti-inflammatory activities of this molecule are dependent upon its modulatory influence on a number of intracellular enzymes including phospholipase A2 (PPA2), COX-2, PKC, 5-lipooxygenase, nitric oxide synthase (NOS), NADPH oxidase and superoxide dismutase (SOD). Further, in vitro studies have shown a modulatory effect of Vitamin directed toward gene expression of factors involved in the pathogenesis of atherosclerosis (such as cytokines, selectins, cyclins, etc.) [22].

Regardless of these numerous evidences randomized clinical trial failed to confirm vitamin E supplementation to be beneficial in reducing the risk of cardiovascular complications [23,24]. However, American Heart Association recommends taking balanced diet rich in vitamin E for the better health of heart. Research has also shown that the oxidation and inflammation induced by CS in animals and cells can be reduced by antioxidants [25,26]. Likewise, the Food and Nutrition Board of the National Academy of Sciences has established a higher recommended dietary allowance (RDA) of vitamin C for smokers (over 200 mg/day versus the recommended 90 mg/day for non-smokers). Vitamin C has been shown to act as a dual negative modulator of oxidative stress (as a hydrophilic antioxidant) and production of lymphocytes and cytokines. Vitamin C also inhibits the activity of phagocytes and similarly to vitamin E, the expression of a number of cell adhesion molecules in monocytes [27]. Furthermore, vitamin C seems to prevents histamine release and increases the detoxification of histamine [28]. Inasmuch active and passive smoking is associated with dysfunction of vascular endothelial physiology [29–36] in a causative and dose dependent way [37] that is largely related to the content of reactive oxygen species (ROS) [31,38,39] and pro-inflammatory activity [39,40]. Cigarette smoke (CS) increases the risk of silent cerebral infarction (SCI) [41] and stroke by approximately 50% [42,43] due to its pro-coagulant and atherogenic effects [44,45] and is currently considered a major public health challenge accounting for over 400,000 deaths/year in US alone. Several studies have shown that chronic smokers suffer from antioxidant shortages caused by increased anti-oxidative mobilization that is evoked by CS [46–48]. Similarly to vitamin E, despite the positive in vitro results, vitamin C-based antioxidant therapies show contradicting outcomes in most clinical trials. Therefore, arguing for or against the prophylactic and/or therapeutic use of antioxidants remains a challenging conundrum.

In contrast to other antioxidants Co-enzyme Q10 – CoQ(10) inhibits both the initiation and the promulgation of lipid and protein oxidation and regenerates vitamin E, thereby further inhibiting the propagation steps of oxidative damage to cellular components. In addition, CoQ(10) seems to prevent the oxidation of mitochondrial DNA and LDL and its use is suggested (among others) as mitochondrial medicine in cardiovascular disorder especially in heart failure. CoQ(10) has also been recommended in hypertension and statin myopathy [49] based on the fact that, declined CoQ(10) levels may impair production of bioenergetics molecules (e.g., ATP), thus leading to increase oxidative stress and dysfunction of cardiac muscle resulting in heart failure [50]. Based on these premises from a number of in vitro and in vivo studies, CoQ(10) seems to possess anti-inflammatory, anti-nociceptive and anti-angiogenic activities and several pathological disorders on top of CVD (including Parkinson’s and Huntington’s diseases) seem to be responsive to CoQ(10) treatment [51,52]. However, a number of clinical trials on CoQ(10) have been published with contradictory results and failed to substantiate any conclusion on the beneficial role of Co-Q10 in primary prevention of cardiovascular disease [53] while other trials claim that long term CoQ(10) adjunct therapy is effective in the treatment of chronic heart failure and consequent cardiovascular complications [54].

α-Lipoic acid (ALA) is a powerful natural antioxidant and essential for the aerobic metabolism and is involved in the recycling of other antioxidants in the body (including vitamins C, E and glutathione) by acting as regenerative substrate. It is commonly present in almost all foods, especially heart, kidney, liver, spinach, broccoli, and yeast. ALA (and its active reduced form, dihydrolipoic acid - DHLA), has been shown to counteract oxidative stress by quenching a variety of ROS and preserve blood-brain barrier (BBB) integrity [55–57]. Differently from vitamin E and C, ALA is amphipathic in nature, thus can act both in lipid and aqueous solutions. In addition to ROS scavenging, ALA has been shown to modulate blood lipid, protect against LDL oxidation and reduce hypertension. These characteristics suggest a potential use for the treatment and/or prevention of CVD as well as other disorders including diabetes, cancer, neurodegenerative, and autoimmune diseases [58–61]. However, despite the numerous studies there is no unanimous consensus on a number of therapeutic parameters including dosage, dose frequency and form of administration.

Glutathione (GSH) is the major endogenous cellular antioxidant which directly participated in the neutralization of free radicals and reactive oxygen compounds and maintains exogenous antioxidants (including vitamins C and E) in their reduced (active) form. Glutathione also plays a central role in the regulation of the nitric oxide cycle, DNA synthesis and repair, protein synthesis, and enzyme activation [62–64]. As a result, the use GSH supplements have been postulated as a useful approach to counteract and/or prevent CVD and stroke, protect the lining of the arteries and reduce LDL oxidation and lower cholesterol by naturally inhibiting its production in the liver [65,66]. However, clinical trials focused on assessing the effects of oral GSH supplementation on systemic oxidative stress did not reveal any significant benefit. The discrepancy between in vivo studies in rodents (rats) and humans could be possibly due to the relative inability of the latter to absorb GSH through the intestinal tract. In fact, while some experimental evidence have suggested an increases in blood and/or intracellular levels of GSH through oral supplementation using reduced GSH in vivo [67]; the absorption of GSH in humans has not been equally confirmed [68,69]. The problem of GSH absorption in human could be complicated by the fact that the human gastrointestinal tract contains substantial amounts of γ-glutamyltransferase (GGT) which recycles GSH precursors and may prevent the oral absorption of intact glutathione itself.

Resveratrol is a natural plant-derived polyphenol which is found in abundance in the skin of grapes, mulberries, raspberries, and blueberries which seems to exert diverse positive biological effects and is currently considered a hot topic in numerous animal and human studies. Anticancer, anti-inflammatory, anti-aging, anti-diabetic and beneficial cardiovascular effects of resveratrol have been reported [70–72] although inconsistently. Not to mention, resveratrol has been shown to have protective effect on the BBB during ischemia [73,74] and in recent years (2003) resveratrol was discovered to be a small molecule activator of Sirtuin 1 (SIRT1), a protein whose activity has been linked to longevity. However, as for now, clinical evidence of these beneficial effects of resveratrol in humans is still lacking. For example, there is no confirmation that resveratrol can benefit patients who already suffer of heart disease [75,76]; although some data support a possible beneficial effect on diabetes [77]; the positive effect on cancer are inconsistent as well [78]. As for the metabolic effects of resveratrol, the clinical data are also inconclusive [79]. Finally, the hoped antiaging effect of resveratrol still remains unclear and yet to be proved [80].
